# Bursting with Randomness: A Simple Model for Stochastic Control of Gene Expression

**DOI:** 10.1371/journal.pbio.1001622

**Published:** 2013-08-06

**Authors:** Richard Robinson

**Affiliations:** Freelance Science Writer, Sherborn, Massachusetts, United States of America

If a gene's promoter were a light switch, you'd probably call an electrician. That's because rather than simply turning on and off in a limited and predictable way, many genes—whose expression is controlled by their promoters—are expressed in bursts, with expression fluctuating randomly over time.

**Figure:**
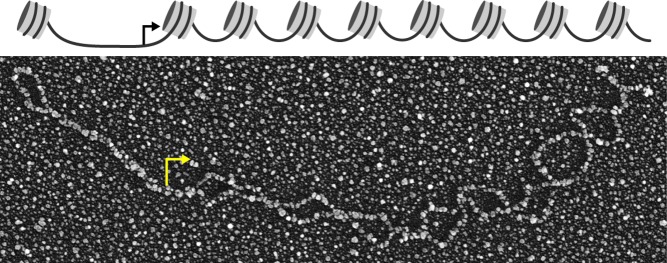
Analyzing the nucleosome configuration of single gene molecules by electron microscopy.

What is the molecular basis of this transcriptional bursting? Previous work has suggested, but not directly demonstrated, that gene promoters can assume alternative structural configurations, raising the possibility that the promoter might randomly flip among these alternatives. Transcription requires that the polymerase machinery gain access to the promoter, and that access can be inhibited when DNA is tightly wrapped around histone proteins to form nucleosomes, the basic protein–DNA unit of structure within chromosomes. Researchers have speculated that these alternative promoter configurations might be linked to the number and distribution of nucleosomes within the promoter, but there has been no proof.

The best way to test that hypothesis is by direct observation, and that is what Christopher Brown, Hinrich Boeger, and colleagues set out to do. Their work confirmed that the promoter of *PHO5*, a well-studied yeast gene, does indeed adopt alternative configurations based on nucleosome distribution. And they went further, examining the probability distribution of those nucleosomes and constructing a mechanistic model that accounts for that distribution, and hence the phenomenon of transcriptional bursting.

The key to their study was to chemically “freeze” the promoter regions of multiple *PHO5* genes, and then use the electron microscope to determine the precise configuration of each. The gene's promoter includes three nucleosome binding sites, and they were able to tabulate the relative frequency of each of the eight possible binding states (i.e., occupied-occupied-occupied, occupied-occupied-empty, etc.).

Next, they built a mathematical probability model to test various assumptions about the transitions among the binding states. They distinguished three kinds of transitions: assembly of a nucleosome on a binding site, disassembly and removal, or sliding of a nucleosome from one site to another. They found that assembly and disassembly alone accounted for the number of observed nucleosomes, but that sliding was needed to account for their distribution. In particular, it appeared that sliding out of the middle position toward the ends was common. Further, the best fit with the data came when disassembly at the first position occurred only when the second position was unoccupied.

Since transcription is maximally repressed in the fully occupied state and maximally permitted in the fully unoccupied state, the stochastic transitions among these and the intermediate states suggest that transcription of the *PHO5* gene occurs in random bursts, the authors concluded. Further experimentation and modeling allowed them to predict that binding states in which the middle position was unoccupied were the most conducive to transcription. But the transcription rate was not maximal in all conducive states; other, as-yet unidentified factors presumably play a role in determining the degree of transcription for a given conducive state.

The model the authors developed provides a structural basis for transcriptional bursting consistent with a large body of data. While other models might also explain that data, none do so as simply and with as few “working parts” as theirs—generally a sign that a model is pointing in the right direction.

**Brown CR, Mao C, Falkovskaia E, Jurica MS, Boeger H (2013) Linking Stochastic Fluctuations in Chromatin Structure and Gene Expression. doi:10.1371/journal.pbio.1001621**

